# Relevance of pharmacogenetic analyses and therapeutic drug monitoring of antidepressants for an individualized treatment of peripartum psychopathology

**DOI:** 10.1097/YIC.0000000000000520

**Published:** 2023-11-20

**Authors:** Anna Colombo, Rita Cafaro, Ilaria Di Bernardo, Marta Mereghetti, Lucia Cerolini, Luca Giacovelli, Federica Giorgetti, Simone Vanzetto, Nicolaja Girone, Valeria Savasi, Irene Cetin, Emilio Clementi, Monica Francesca Bosi, Caterina Adele Viganò, Bernardo Dell’Osso

**Affiliations:** a Department of Mental Health and Addiction, ASST Fatebenefratelli-Sacco; b Department of Biomedical and Clinical Sciences ‘Luigi Sacco’, University of Milan; c Department of Woman, Mother and Neonate, Luigi Sacco Hospital, ASST Fatebenefratelli-Sacco; d Department of Woman, Mother and Neonate, Buzzi Children Hospital, ASST Fatebenefratelli Sacco; e Department of Laboratory Medicine and Diagnostic Imaging, Unit of Clinical Pharmacology, Luigi Sacco Hospital, ASST Fatebenefratelli-Sacco; f Department of Health Sciences, ‘Aldo Ravelli’ Center for Neurotechnology and Brain Therapeutic, University of Milan, Milan, Italy; g Department of Psychiatry and Behavioral Sciences, Bipolar Disorders Clinic, Stanford University, Stanford, California, USA

**Keywords:** antidepressants, pregnancy, psychiatric symptoms during pregnancy, safety of antidepressants

## Abstract

**Objective:**

Psychiatric disorders burden the peripartum period, often requiring psychopharmacological treatment, including antidepressants. Efficacy and tolerability of antidepressants are influenced by the physiological changes of the peripartum and individual metabolic profiles, which in turn can be modified by pregnancy. The objective of this study is to assess the relationship between antidepressants’ pharmacokinetic profiles during pregnancy and individual metabolic profiles, along with the efficacy of the treatment.

**Methods:**

In total 87 outpatients with diagnoses of bipolar disorder, major depression, anxiety, obsessive-compulsive disorder and post-traumatic stress disorder who required antidepressant treatment during pregnancy were recruited. Genotyping analysis of hepatic cytochrome P450 (CYPs) individual isoforms was performed. Antidepressants’ blood concentrations and psychometric assessments were collected at five time points. Antidepressants’ cord blood concentrations were assessed at birth.

**Results:**

Sertraline showed greater stability in plasma concentrations and a lower placental penetrance index. Most of the antidepressants’ concentrations below the therapeutic range were found in women with an extensive/ultrarapid metabolic profile. Antidepressants mainly metabolized by CYP2C19 were less frequently below the therapeutic range compared with antidepressants metabolized by CYP2D6.

**Conclusions:**

Pregnancy modulates cytochrome activity and drugs’ pharmacokinetics. Genotyping analysis of CYPs isoforms and therapeutic drug monitoring might be used to guide clinicians in a well-tolerated treatment of psychiatric symptoms in pregnant women.

## Introduction

Depressive disorders during pregnancy and postpartum are common and disabling conditions. Depression affects women during pregnancy with an estimated prevalence of 10–20% (Van Niel and Payne, 2020). During the first 3 months after delivery, almost 20% of all women report depressive symptoms, and around 7% of these reach the criteria for a major depressive episode. Also, anxiety disorders (3%), specific phobias (6%) and obsessive-compulsive disorders (OCD, 3%) are common disorders that can affect pregnant women (Fawcett *et al.*, 2019). Several studies identify a familiar or personal history of psychiatric disorders as a main predictor for developing overt psychiatric symptoms during pregnancy (Fisher *et al.*, 2012; Hübner-Liebermann *et al.*, 2012). Räisänen *et al*. (2014), in their sample of women who received a diagnosis of major depressive disorder during pregnancy, found a prevalence of previous major depressive episodes of around 50% (Räisänen *et al.*, 2014). Nonetheless, pregnancy alone can be considered an at-risk state for the development of newly onset psychiatric conditions (Guintivano *et al*., 2018). Given the high risk of experiencing psychiatric symptoms during pregnancy and their negative influence on pregnancy itself, the need for psychopharmacological therapy to treat these conditions is one of the major concerns for both psychiatrists and gynecologists. Guidelines recommend starting a pharmacological treatment with an effective drug, possibly in monotherapy, used at the lowest effective dose. Selective serotonin reuptake inhibitors are considered first-line choice in pregnant women suffering from depressive disorders, obsessive-compulsive disorders and anxiety disorders (Rubinchik *et al*., 2005; Uguz, 2015; Vigod et al., 2016). Among selective serotonin reuptake inhibitors, sertraline and citalopram are recommended for their safety profiles (Myles *et al.*, 2013).

Physiological changes occurring during pregnancy determine changes in the absorption, distribution, metabolism and excretion of medications, consequently affecting their bioavailability. This is especially the case of CYP3A4, CYP2A6, CYP2D6 and CYP2C9 enzymes, whose overexpression during pregnancy is responsible for the increased metabolism of some psychotropic drugs, suggesting that many pregnant women on antidepressants may require dose increases to maintain euthymia (Deligiannidis and Freeman, 2014; Feghali et al., 2015). Patient’s therapeutic response might also be influenced by polymorphisms of genes encoding drug receptors, drug transporters and enzymes involved in drug metabolism. However, the clinical response to antidepressants is mainly influenced by the CYP polymorphisms, which could be classified into four phenotypes: the ultrarapid metabolizers (UMs), the normal/extensive metabolizers (EMs), the intermediate metabolizers (IMs) and poor metabolizers (PMs). Normal/EMs have normal enzymatic capacity and carry homozygous alleles. Poor metabolism is due to the presence of two nonfunctional alleles or deletions of entire genes, so that PM do not possess the active enzyme. IMs carry genotypes connected with substantially reduced but not abolished enzymatic capacity. Finally, UMs often carry more than one extra functional gene, connected with higher enzymatic capacity (Zhou *et al.*, 2010). The CYP enzymes mainly involved in the metabolism of antidepressants involved in this study are shown in Supplementary material, Supplemental digital content 1, http://links.lww.com/ICP/A124. Sertraline, escitalopram and citalopram are mainly metabolized by CYP2C19, whereas paroxetine, fluoxetine, venlafaxine and duloxetine are mainly metabolized by CYP2D6.

Therapeutic drug monitoring (TDM) is the clinical practice of measuring blood concentrations of specific drugs at designated intervals to maintain constant concentrations in a patient’s bloodstream (Arfman *et al.*, 2020). For the majority of medications, it is usually unnecessary to employ TDM; however, given the many factors possibly influencing drug concentrations during pregnancy and postpartum and the harmful consequences of uncontrolled drug exposure on both women and their children (i.e. poor psychiatric symptoms control, possible side effects on the newborn, etc.), TDM may be useful.

In light of these considerations, the aim of this study was to implement TDM in patients taking antidepressants during pregnancy and postpartum, to study the pharmacokinetic profiles of these drugs, their safety and their psychopathological correlates. Moreover, pharmacogenetic analyses were used to identify CYP polymorphisms and their influence on drug blood concentrations.

## Material and methods

### Participants

Eighty-seven (1) pregnant women with (2) a psychiatric diagnosis according to the Diagnostic and Statistical Manual of Mental Disorders, 5th edition (Diagnostic and statistical manual of mental disorders: DSM-5, 2013) and (3) an ongoing treatment with an antidepressant, which performed (4) at least one peripheral blood sampling of plasma drug concentrations, were included in the study. Subjects were enrolled between 2011 and 2019 at the specialized outpatients service ‘Centre for the Treatment of Depressive Disorders’ of the ASST Fatebenefratelli–Sacco, Sacco University Hospital (Milano, Italy). Enrolled women were either referred to the clinic before or during their pregnancy; therefore, analyses and assessments performed in the study are consistent with their time of entry in the study, according to the phase of pregnancy. Women (1) affected by psychotic disorders, (2) taking any other concomitant psychopharmacological therapy (with the exception of benzodiazepines up to an equivalent daily dose of 0.5 mg of alprazolam) or who (3) underwent any change in the psychopharmacological therapy administered during the time of follow-up, were excluded from the study. Although the use of different molecules (i.e. generic vs. brand) might provide slight differences in plasma drug concentrations, these differences should be in the range of those accepted by bioequivalence studies on generic drugs, and, once included in the study, subjects were instructed to maintain the same drug during the study, therefore limiting intraindividual fluctuations due to changes in bioequivalence. The following socio-demographic and clinical variables were collected: age, educational level, occupation, ethnicity, psychiatric diagnosis, comorbidity with other psychiatric disorders, family history of psychiatric disorders, ongoing antidepressant, concomitant therapies, organic comorbidities (medical conditions requiring long-term medications), ongoing psychotherapeutic treatment, cigarette smoking and alcohol consumption, number of life-time miscarriages or voluntary interruptions of pregnancy. The study was performed in accordance with the principles of the Declaration of Helsinki regarding medical research in humans, and it satisfied local research ethical requirements. All patients provided written informed consent before undergoing any study procedure.

### Time points

Chosen time points were: (1) the first trimester of pregnancy (within 13 gestational weeks + 1 day = T1); (2) the second trimester of pregnancy (from 13 weeks + 2 days to 26 weeks + 2 days = T2); (3) the third trimester of pregnancy (from 26 weeks + 3 days to birth = T3); (4) birth (within 2 weeks from birth = T4) and (5) the postpartum period (at least 2 months after birth = T5). At each time-point peripheral blood sampling of plasma drug concentrations was carried out, along with a psychometric assessment. At T4, when possible, cord blood sampling of plasma drug concentrations was obtained. This analysis was not performed on women who either did not give consent for the procedure or did not give birth at Sacco University Hospital.

### Psychometric assessment

Psychometric assessment of anxiety and depression symptoms was obtained through the administration by adequately trained psychiatrists of (1) the Hamilton Anxiety Rating Scale (Ham-A), (2) the Hamilton Depression Rating Scale (Ham-D) and (3) the Montgomery-Asberg Depression Rating Scale (MADRS) (Hamilton, 1959, 1960; Montgomery and Asberg, 1979). Remission was defined as a reduction of 7 or more at the Ham-A and Ham-D and a reduction of 10 or more at the MADRS.

### Pharmacokinetic analyses

Peripheral blood samples were collected from 8 to 15 h after the last oral drug administration. Cord blood samples were collected immediately after birth. Samples were then stored at −20 °C and subsequently analyzed with chromatographic and mass spectrometric techniques developed and validated at the Pharmacokinetics and Pharmacogenetics Laboratory of the O.U. of Clinical Pharmacology of the ‘L. Sacco’ Hospital. The lower limit of quantification was 5 ng/mL for all the analyses. The performance of these methodologies was tested during each analytical activity using both internal quality controls and as part of the Laboratory of the Government Chemist Standard Proficiency Testing Schemes for Psychoactive Drugs. In accordance with the Arbeit gemeinschaft für Neuropsychopharmakologie und Pharmakopsychiatrie guidelines (Hiemke *et al.*, 2018), the plasma concentration considered as therapeutic ranges are sertraline 10–150 ng/ml; escitalopram 15–80 ng/mL; citalopram 50–110 ng/mL; paroxetine 20–65 ng/mL; fluoxetine 120–500 ng/mL; venlafaxine 100–400 ng/mL; duloxetine 30–120 ng/mL.

#### Weighted plasma concentrations

Weighted plasma concentrations per administered dosage (C/D = ng/mL: mg/die) were calculated at each time point, either from the mother or the cord blood, dividing the plasma drug concentrations per women’s daily drug intake at the time of sampling to eliminate the potential confounding factor given by nonuniform drug dosages between women (Paulzen *et al.*, 2017a).

#### Placental Penetrance Index

The Placental Penetrance Index (PPI) was assessed by dividing the C/D in the cord blood by the C/D at T3 and T4 in the women’s peripheral blood samples.

### Pharmacogenetic analyses

Peripheral blood samples obtained at the first time point available, accordingly with the time of entry in the study and the phase of pregnancy, were also used to determine the genotype of the hepatic CYP isoforms involved in the metabolism of the specific antidepressant taken from each patient, to identify UMs, normal/EMs, IMs and PMs. Women were divided into two groups based on their metabolic profile: PMs/IMs vs. EMs/UMs. DNA was extracted, and CYP isoform polymorphisms were identified through Real-Time PCR with LightSNiPR (TIBMolBiol, Berlin) or TaqManR (Thermo Fisher Scientific, Waltham, Massachusetts, USA) according to the manufacturer’s instructions.

### Statistical analyses

Sociodemographic and clinical variables were compared between women taking different antidepressants using the chi-square test and the Student’s *t*-test for categorical and continuous variables, respectively. The number of plasma drug concentrations in range vs. below therapeutic range was compared through a chi-square test for each antidepressant and at each time point, in the total sample and in the two subgroups of PMs/IMs and EMs/UMs. Among these subgroups of metabolic profiles, comparisons between plasma drug concentrations within vs. below therapeutic range were lastly repeated based on the main metabolic pathway of antidepressants in use (drugs metabolized by CYP2C19 vs. drugs metabolized by CYP2D6). Paired-samples Student’s *t*-test was used to compare plasma drug concentrations and C/Ds at each time-point among women taking the same antidepressant. Finally, psychometric assessment scores were compared among women taking different antidepressants at each time point. Statistical significance was set at *P* < 0.05. The software IBM SPSS Statistics, version 26.0, Armonk, NY: IBM Corp. was used to perform the analyses.

## Results

Sociodemographic and clinical variables of enrolled women are reported in Supplementary material, Supplemental digital content 1, http://links.lww.com/ICP/A124. No significant difference was found between women taking different antidepressants. Given the different time of entry in the study and the subsequently different phase of pregnancy upon entry, the number of peripheral blood samples collected at each time point is presented in Table 1, along with the percentage of plasma drug concentrations within the therapeutic range at each time-point. No women showed plasma drug concentrations above the therapeutic range.

**Table 1:** N° of blood samples by follow-up time and their percentage within therapeutic range

N° of valid blood samples (% TR)	T1	T2	T3	T4	T5	Cord blood sample
Total n°	27	53	53	51	37	43
Sertraline	13 (76%)	30 (86%)	26 (84.6%)	25 (56%)	20 (80%)	24 (25%)
Paroxetine	3 (33.3%)	5 (40%)	5 (40%)	10 (20%)	4 (75%)	7 (0.0%)
Escitalopram	4 (75%)	5 (60%)	8 (50%)	6 (33.3%)	3 (33.3%)	3 (33.3%)
Citalopram	//	2 (100%)	3 (33.3%)	1 (0.0%)	1 (100%)	1 (0.0%)
Fluoxetine	//	1 (100%)	1 (100%)	3 (66%)	//	3 (33.3%)
Venlafaxine	6 (66.7%)	9 (44,4%)	9 (55,6%)	6 (66,7%)	7 (85.7%)	5 (60%)
Duloxetine	1 (0.0%)	1 (0.0%)	1 (100%)	//	2 (0.0%)	//

### Comparison between plasma drug concentrations within and below therapeutic range in the general sample and by metabolic profile subgroups

**Fig. 1:**
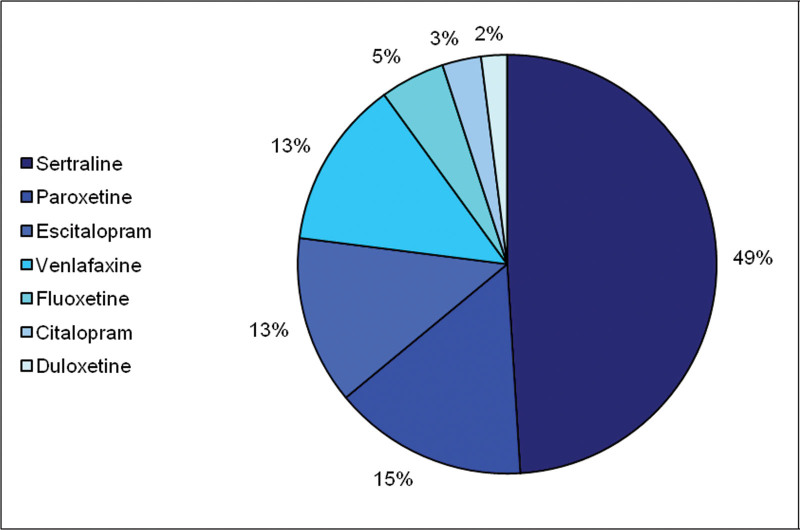
Distribution of antidepressant prescriptions in our sample.

**Fig. 2:**
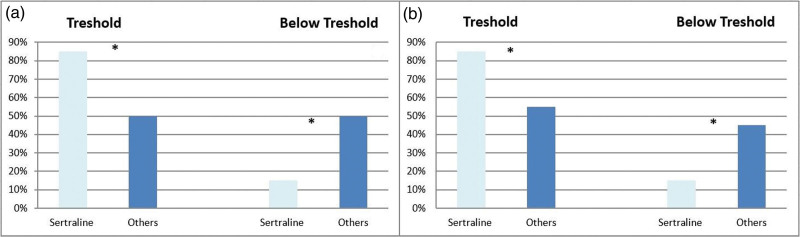
Percentages of subjects within therapeutic ranges of Sertraline compared to other drugs at T2 and T3. (a) Percentages of subjects showing therapeutic ranges of sertraline compared with other drugs at T2; (b) Percentages of subjects within therapeutic ranges of sertraline compared with other drugs at T3. * = P < 0,05.

About half of the participants took sertraline (Fig. 1). Among all antidepressants, sertraline showed a higher percentage of plasma drug concentrations within the therapeutic range both at T2 (*P* = 0.012) and T3 (*P* = 0.007) (Fig. 2). Among EMs/UMs (*N* = 49), sertraline showed the higher percentage of within range plasma drug concentrations at T3 (*P* = 0.017). Sertraline also showed a lower placental penetrance index (PPI = 0.43; Table 2), while it ranged between 0.71 and 0.78 for other antidepressants.

**Table 2:** C/D at T3 and in cord blood, transplacental passage index

Drug	T3 daily dose	T3 mother blood concentrations	Mother C/D	Cord blood concentrations	Infant C/D	PPI
Sertraline	72.67 ± 36.52 (25–200)	27.04 ± 22.75 (2.5–90.7)	0.35 ± 0.22 (0.03–0.85)	7.89 ± 7.80 (2.5–35.5)	0.11 ± 0.1 (0.0–0.38)	0.43 ± 0.26 (0.06–1)
Paroxetine	18.08 ± 4.08 (10–25)	14.17 ± 16.05 (2.5–41.9)	1.08 ± 1.58 (0.13–4.19)	3.61 ± 2.95 (2.5–10.3)	0.22 ± 0.14 (0.13–0.52)	0.76 ± 0.41 (0.08–1)
Escitalopram	10.64 ± 5.42 (5–20)	18.89 ± 21.33 (2.5–68.8)	1.87 ± 2.19 (0.25–6.88)	8.7 ± 6.75 (2.5–15.9)	1.13 ± 0.76 (0.25–1.59)	0.78 ± 0.26 (0.49–1)
Citalopram	20 ± 0.00 (20–20)	49 ± 38.15 (14.9–90.2)	2.45 ± 1.9 (0.75–4.51)	(*) 5.5	(*) 0.28	(*) 0.60
Fluoxetine	22.50 ± 5.00 (20–30)	(*) 346.8	(*) 11.56	87.70 ± 76.91 (2.5–152)	4.39 ± 3.84 (0.13–7.60)	0.71 ± 0.5 (0.13–1)
Venlafaxine	95.46 ± 35.02 (75–150)	146.12 ± 86.68 (37.6–260.1)	1.73 ± 1.03 (0.33–3.27)	154.54 ± 67.46 (78.9–234.7)	1.64 ± 0.84 (0.53–2.46)	0.77 ± 0.23 (0.40–0.98)
Duloxetine	45.0 ± 21.21 (30–60)	(*) 11.2	(*) 0.37	//	//	//

Drug daily dose expressed as mg/die; blood concentrations expressed as ng/mL; the table shows means concentrations ± SD (min-max); C/D: concentration per dosage = ng/mL: mg/die.

PPI was assessed by dividing the C/D in the cord blood per the C/D at T3 and T4 in the women’s peripheral blood samples.

PPI, placental passage index.

### Comparison between plasma drug concentrations within and below therapeutic range by metabolic profile subgroups

Comparing the subgroups of PMs/IMs vs. EMs/UMs, it emerged that 94.1% of plasma concentrations below therapeutic range at T3 concerned the group of EMs/UMs (*P* = 0.006); this datum was confirmed at T4, with 81.3% of plasma concentrations below therapeutic range in the EMs/UMs group (*P* = 0.049).

### Comparison of plasma drug concentrations within and below therapeutic range between antidepressants metabolized by CYP2C19 vs. CYP2D6

When comparing plasma concentrations within vs. below the therapeutic range between drugs mainly metabolized by CYP2C19 vs. CYP2D6, we found significant differences among EMs/UMs (*P* = 0.010), but not among PMs/IMs. Specifically, at T2, 80% of plasma drug concentrations in the CYP2C19 group were within the therapeutic range, while in the group of molecules metabolized by CYP2D6, only 25% of plasma concentrations were within the range (*P* = 0.010). Also, at T3, 90% of plasma drug concentrations in the CYP2C19 group were within therapeutic range (*P* = 0.019).

### Comparison of plasma drug concentrations at different time points among group of patients exposed to the same antidepressant through paired-sample Student *t*-tests

For sertraline, the paired-samples Student’s *t*-test of mean plasma drug concentrations at T4 and T5 showed a significant difference (12.01 ± 6.3 vs. 33,5 ± 25.5; *P* = 0.019), confirmed also when comparing mean C/Ds at T4 and T5 (0.16 ± 0.14 vs. 0.37 ± 0.34; *P* = 0.029).

Also, venlafaxine showed a significant difference in plasma drug concentrations at T4 and T5 (138.38 ± 72.9 vs. 262 ± 63.57; *P* = 0.011), which was confirmed when comparing mean C/Ds at T4 and T5 (1.18 ± 0.2 vs. 2.5 ± 0.67; *P* = 0.046).

Neither sertraline nor venlafaxine had significantly different plasma drug concentrations at T1 and T5, nor C/Ds.

No other antidepressant showed significant differences in plasma drug concentrations at different time points.

### Psychometric assessment

From a clinical perspective, scoring of the scales administered at each time-point showed an overall improvement in depressive and anxious symptoms, as shown in Table 3. During the observation period (i.e. from T1 to T5), the percentage of patients who achieved clinical remission according to the psychometric scores increased from 37.9 to 71.9% (HAM-D), from 60 to 92.4% (HAM-A) and from 42.4 to 72.7% (MDRS).

**Table 3:** Psychometric assessment scores by follow-up visits

	HAM-D	HAM-A	MADRS
T1	12.6 ± 9.11 (0–34)	15.5 ± 10.89 (1–39)	13.78 ± 10.55 (1–38)
T2	9.98 ± 8.22 (0–38)	11.93 ± 8.88 (0–44)	11.29 ± 9.50 (0–49)
T3	7.91 ± 6.18 (0–28)	10.68 ± 7.41 (0–38)	8.85 ± 7.75 (0–36)
T5	7.35 ± 6.96 (0–28)	10.04 ± 8.83 (0–40)	8.15 ± 7.80 (0–36)

Scores are presented as mean ± SD (min-max).

HAM-A, Hamilton Anxiety scale; HAM-D, Hamilton Depression scale; MADRS, Montgomery-Asberg Depression Rating Scale.

## Discussion

In the present study, through the application of therapeutic drug monitoring, we aimed to evaluate the efficacy and safety of antidepressants during pregnancy, a period of important physiological changes to the metabolism of medications, in which both underdosing and overdosing of drugs could have severe consequences on women and their children.

Our main results showed that, among all antidepressants, sertraline had the higher percentage of plasma drug concentrations within the range at T2 and T3 in the general sample, and at T3 in the subgroup of EMs/UMs. Sertraline also showed a lower placental penetrance index. The majority of plasma drug concentrations below the therapeutic range were found at T2 and T3 and regarded patients with an extensive/ultrarapid metabolic profile and drugs metabolized by CYP2D6. Of all antidepressants, only sertraline and venlafaxine showed plasma drug concentrations significantly different between T4 and T5, but not between T1 and T5.

The great stability in plasma concentrations found for sertraline is in line with previous data from the literature. This stability may be motivated by the inhibition of the metabolic activity of CYP2C19, which could help minimize the difference between PMs and EMs (Sit *et al.*, 2008; Westin *et al.*, 2017). The inhibition of CYP2C19 might also explain why sertraline showed greater stability in plasma drug concentrations in the group of EMs/UMs.

Moreover, sertraline also showed a lower placental passage index compared with other antidepressants, even lower than reports in the literature (Paulzen *et al.*, 2017b). PPI value assumes an important role in the safety evaluation of chosen antidepressants, given that a significant direct proportionality is frequently observed between the maternal plasma concentrations and the fetus-newborn ones (Paulzen *et al.*, 2017a; Paulzen *et al.*, 2017b).

When studying metabolic profiles of enrolled women (i.e. identifying PMs/IMs and EMs/UMs), we found that the majority of below-range plasma drug concentrations regarded subjects with an extensive/ultrarapid metabolic profile, regardless of taken antidepressant and phase of pregnancy. To evaluate the interconnection between changes in metabolic profiles due to pregnancy and plasma drug concentrations, we compared the plasma concentration of antidepressants metabolized by CYP2C19 (sertraline, escitalopram and citalopram) with those metabolized by CYP2D6 (paroxetine, fluoxetine, venlafaxine and duloxetine). We found plasma drug concentrations significantly less frequently below the therapeutic range in the first group, in accordance with the well-known effects of pregnancy, which slow down the metabolic activity of CYP2C19 and accelerate the activity of CYP2D6 (Sit *et al.*, 2008; Westin *et al.*, 2017).

The effects of the physiological changes of pregnancy were also evident considering the trend of plasma concentrations during pregnancy at delivery and in postpartum, showing that, for the same dosage, plasma concentrations at T5 (the postpartum period, i.e., after the remission of pregnancy physiologic changes) were significantly greater than at the end of pregnancy (i.e. T4), when there is the maximum plasmatic volume expansion and the effects on metabolic profiles are still ongoing. This result was confirmed for sertraline and venlafaxine, both showing higher plasma drug concentrations and C/Ds at T5 compared with T4. In fact, as it would be expected, in the postpartum period, the paraphysiological changes of pregnancy are considered resolved, the plasmatic volume is reduced, and the drug is less hemodiluted. In confirmation of these considerations, no significant differences were observed between either plasma drug concentrations or C/Ds of sertraline and venlafaxine at T1 and T5, since in the first trimester of pregnancy, gestational changes are not in progress yet and therefore the conditions are more similar to the postpartum period.

This data, however, differs from what was found in a previous study (Westin *et al.*, 2017), in which an opposite trend is reported for sertraline, while no variation was found in venlafaxine plasma concentrations between the third quarter and the postpartum. However, this study conducted on Norwegian birth registers did not consider the oral dosage of the drug taken, introducing a potential confounding factor, which in the present work was excluded by comparing C/Ds.

Overall, depressive and anxious symptoms improved during the follow-up, as measured by psychometric scales administered at each follow-up time point. This data confirms the need for psychopharmacological therapy for the management of anxious and depressive symptoms in the context of a close follow-up during pregnancy, which does not constitute a confounding factor in this study. Although many studies focus on evaluating the safety of antidepressants during pregnancy, limited data exist on antidepressant treatment efficacy during pregnancy. Clinical evidence recommends that increases or adjustments to antidepressant doses might be necessary during pregnancy, therefore highlighting how more stable plasma concentrations could improve the manageability of treatment. However, in our study, subjects treated with all antidepressants provided similar results at psychometric evaluations of symptoms, possibly suggesting maintenance of long-term efficacy regardless of fluctuations in plasma drug concentrations when these happen within therapeutic ranges.

Our results, albeit preliminary, underline that carrying out therapeutic drug monitoring of antidepressants during pregnancy might be a useful tool for the careful monitoring of both the efficacy and safety of the chosen pharmacotherapy. Moreover, our study highlights the importance of taking into consideration both the metabolic profile of the chosen drug (considering the effects of pregnancy on the hepatic metabolism) and the patient’s individual metabolic profile when choosing the antidepressant. When therapeutic drug monitoring is not possible, sertraline showed good stability in plasmatic concentrations regardless of the individual’s metabolic profiles and phases of pregnancy. Along with previous studies from our group, which highlighted the safety of antidepressant use during pregnancy as measured with maternal and neonatal outcomes, this study enriches the literature on this controversial topic, aiding clinicians in making the best therapeutic choices for their patients (Perrotta *et al.*, 2019; Colombo *et al.*, 2020).

However, one main limitation of our work lies in the great asymmetry with respect to antidepressants taken by enrolled women. Moreover, being a naturalistic sample, it was not possible to have a control sample consisting of women with anxiety or depressive disorders in pregnancy not under pharmacological treatment. Furthermore, it was not possible to control the sample for clinical and sociodemographic characteristics such as exposure to cigarette smoke (which occurred in 11 cases), alcohol (which occurred in only one case), benzodiazepines taken at a dosage higher than alprazolam 0.5 mg per day (in only one case) and concomitant medical therapies.

### Conclusion

Anxiety and depressive disorders in peripartum represent significant public health problems, for which pharmacological treatment has to be accurately evaluated. Studies on the safety of antidepressants’ use during pregnancy have often not been able to distinguish in what proportion pharmacological treatment and psychopathology may be influenced by the obstetric-gynecological changes of pregnancy. The results of this work, albeit still in a preliminary way, support the usefulness of the identification of individual’s metabolic profiles and of therapeutic drug monitoring of plasmatic concentrations during pregnancy, therefore directing the choice of antidepressants toward the most effective and well-tolerated treatment with a view to individualizing treatment in the field of precision medicine.

## Supplementary Material


