# Supplementary material for: Relevance of pharmacogenetic analyses and therapeutic drug monitoring of antidepressants for an individualized treatment of peripartum psychopathology
Source: Int Clin Psychopharmacol. 2023 Nov 20;39(2):106–12. doi: 10.1097/YIC.0000000000000520 (PMC10833183; doi:10.1097/YIC.0000000000000520)
Supplement: Supplementary file 1 [file icp-39-106-s001.pdf]

SUPPLEMENTARY MATHERIAL

P450 enzymes mainly involved in the metabolism of antidepressants prescribed in our sample

**Antidepressant**	**CYP enzymes**
Sertraline	CYP2C19<br>CYP2B6
Paroxetine	CYP2D6
Escitalopram	CYP2C19<br>CYP2D6<br>CYP3A4
Citalopram	CYP2C19<br>CYP2D6<br>CYP3A4
Fluoxetine	CYP2D6<br>CYP2C9
Venlafaxine	CYP2D6
Duloxetine	CYP1A2<br>CYP2D6

Main socio-demographic and clinical characteristic of our sample

Variable	Frequency [N(%)]
**Total sample**	87 (100)
**Nationality**	
Italian	69 (79.3)
Other	18 (20.7)
**Marital Status**	
Married	37 (42.5)
Unmarried	39 (44.8)
Single	3 (3.5)
Unknown	8 (9.2)
**Education**	
Primary School	0 (0)
Secondary School	10 (11.5)
High School	33 (37.9)
University	28 (32.2)
Unknown	16 (18.4)
**Occupation**	
Employed	35 (40.2)
Unemployed	8 (9.2)
Unknown	44 (50.6)
**Diagnosis**	
Major depressive disorder	39 (44.8)
Anxiety disorder	36 (41.4)
Obsessive-compulsive disorder	7 (8.0)
Bipolar disorder type II	4 (4.6)
Post-traumatic stress disorder	1 (1.2)

**Psychiatric comorbidity**	
Yes	21 (24.1)
**Organic comorbidity**	
Yes	13 (14.9)
**Other medications**	
Non psychiatric	22 (25.3)
Benzodiazepines	11 (12.6)
S-adenosylmethionine infusive therapy	5 (5.7)
**Active tobacco use**	
Yes	11 (12.6)
**Active alcohol use**	
Yes	1 (1.2)
**Current antidepressant**	
Sertraline	43 (49.5)
Paroxetine	13 (14.9)
Escitalopram	11 (12.6)
Citalopram	3 (3.5)
Fluoxetine	4 (4.6)
Venlafaxine	11 (12.6)
Duloxetine	2 (2.3)
**Metabolic profile**	
Poor	5 (7)
Intermediate	17 (24)
Extensive	39 (61,9)
Ultra rapid	10 (7,1)
Missing	16
